# Combined Tissue-Fluid Proteomics to Unravel Phenotypic Variability in Amyotrophic Lateral Sclerosis

**DOI:** 10.1038/s41598-019-40632-4

**Published:** 2019-03-14

**Authors:** Emanuela Leoni, Michael Bremang, Vikram Mitra, Irene Zubiri, Stephan Jung, Ching-Hua Lu, Rocco Adiutori, Vittoria Lombardi, Claire Russell, Sasa Koncarevic, Malcolm Ward, Ian Pike, Andrea Malaspina

**Affiliations:** 1 Proteome Sciences R&D GmbH & Co. KG, Altenhöferallee 3, Frankfurt am Main, 60438 Germany; 2 Proteome Sciences plc, Hamilton House, Mabledon Place, London, WC1H 9BB UK; 3 Centre for Neuroscience and Trauma, Blizard Institute, Queen Mary University of London, 4 Newark Street, London, E1 2AT UK

## Abstract

The lack of biomarkers for early diagnosis, clinical stratification and to monitor treatment response has hampered the development of new therapies for amyotrophic lateral sclerosis (ALS), a clinically heterogeneous neurodegenerative disorder with a variable site of disease initiation and rate of progression. To identify new biomarkers and therapeutic targets, two separate proteomic workflows were applied to study the immunological response and the plasma/brain proteome in phenotypic variants of ALS. Conventional multiplex (TMT) proteomic analysis of peripheral blood mononuclear cells (PBMCs) was performed alongside a recently introduced method to profile neuronal-derived proteins in plasma using brain tissue-enhanced isobaric tagging (TMTcalibrator). The combined proteomic analysis allowed the detection of regulated proteins linked to ALS pathogenesis (RNA-binding protein FUS, superoxide dismutase Cu-Zn and neurofilaments light polypeptide) alongside newly identified candidate biomarkers (myosin-9, fructose-bisphosphate aldolase and plectin). In line with the proteomic results, orthogonal immunodetection showed changes in neurofilaments and ApoE in bulbar versus limb onset fast progressing ALS. Functional analysis of significantly regulated features showed enrichment of pathways involved in regulation of the immune response, Rho family GTPases, semaphorin and integrin signalling. Our cross-phenotype investigation of PBMCs and plasma/brain proteins provides a more sensitive biomarker exploratory platform than conventional case-control studies in a single matrix. The reported regulated proteins may represent novel biomarker candidates and potentially druggable targets.

## Introduction

Amyotrophic lateral sclerosis (ALS), a fatal neurodegenerative disorder, exhibits a highly personal clinical profile from onset with remarkable heterogeneity in the initial site of neurological impairment, rate of progression, and extent of involvement of secondary sites of neuromuscular weakness^1^. Remarkably, end-stage disease characterized by respiratory failure requiring ventilation can be reached within a few months from the first signs of the disease or in up to two decades^2^. Among those clinical features that have emerged as strong prognostic determinants, bulbar function involvement (B-ALS) and signs of frontotemporal pathology appear in association with a rapidly developing disease compared to limb-onset ALS (L-ALS)^3^.

Currently, there are no established blood- or cerebro-spinal fluid (CSF)-based biomarkers which can outperform clinical observation to provide an early diagnosis, an accurate prediction of disease progression or help in the clinical stratification of ALS phenotypic variants. Recently, the measurement of neurofilaments (Nf), key structural components of neurons and axons released into blood upon neurodestruction, has proved very effective in the early detection of ALS cases with different rates of disease progression^4^. While this approach is very informative on the pattern of disease progression, there is the need of a more granular discrimination of the clinical variants of ALS, where B-ALS and L-ALS phenotypes carry a significantly different prognosis and associate themselves with a variable speed of disease progression.

The immunological response to the development of neurodegeneration has become one of the main sources of informative biomarkers in ALS with changes in circulating T-cells and humoral responses being reported as systemic readouts of microglial activation in brain^5,6^. While increased levels of T lymphocytes and dendritic cells are detected in brains from ALS patients^5^, reduced levels of T regulatory cells have been reported in blood from ALS patients, particularly in rapidly-progressing ALS patients^6^.

Among the best target for immunoprofiling, peripheral blood mononuclear cells (PBMCs) have recently been found to be a particularly good source of markers linked to oxidative stress and phosphorylation status, as well as to transcriptional and epigenetic regulation^7,8^. More directly related to the underlying disease processes in ALS, levels of dipeptide repeats, by-products of the C9orf72 gene expansion described in familial ALS, were found to be elevated in PBMCs as well as in CSF from ALS patients^9^. The use of PBMCs as a surrogate for plasma in biomarker discovery was evaluated and found to overcome the challenges of high-abundant plasma proteins masking changes in the less abundant proteins which are mostly below the limit of detection in unbiased mass spectrometry methods^10^. On this basis, PBMCs might represent a useful source of molecules to enhance disease stratification in ALS using TMT multiplex quantitative mass spectrometry (MS).

Mass spectrometry using MALDI-TOF and SELDI has been employed in a range of proteomic studies in ALS driving the identification of candidate disease biomarkers in plasma and CSF^11–13^. While these studies have had the merit of opening promising avenues of investigation, their potential for new biomarkers discovery has been hampered by two substantial limitations: (i) the presence of highly abundant plasma proteins (albumin and IgGs among others) limiting the detection of lower abundand proteins and (ii) the lack of specificity in the selection of disease-related biological signals when a pathological state like ALS characterised by such a wide clinical and biological heterogeneity is compared to a healthy state^14–16^.

Recently, we developed a novel tissue-enhanced proteomic workflow called TMTcalibrator for quantifying low abundant proteins in body fluids^17^. Using isobaric mass tags (TMT 10plex) it is possible to mix and quantify up to 10 different samples in a single mass spectrometry run. This provides an opportunity to combine tissue digests, rich in disease-related proteins, with plasma samples where leakage from affected tissue like brain allows biomarkers to be released although generally at very low concentrations. By depletion of higly abundant plasma proteins (albumin and IgGs) and by selecting an appropriate excess of tissue-derived proteins, it is possible to boost the signal intensity of tissue-derived proteins in the body fluid so that they are selected for MS analysis. We have recently designed a TMTcalibrator plasma/PBMCs experiment to study the rate of ALS progression as the main comparator in individuals and animal models of ALS^18^. This novel methodological approach has shown biological features like senescence, glucose-uptake metabolism and RHO GTPase along with a range of immunological signals as the main drivers of speed of disease progression.

In this study, we have addressed the limitations mentioned above and used well-characterized B-ALS and L-ALS test cohorts which are also categorised according to speed of disease progression. The investigation combines unbiased immune cell proteomics on the one hand and brain tissue-enhanced plasma proteomics on the other. Conventional immunodetection of ALS-representative proteins confirmed the expression and regulation in the phenotypic dimensions of proteins identified using our combined proteomics. Protein expression and functional analysis in each study showed consistently regulated features in peripheral cells and fluid which hold a strong potential as disease biomarkers in ALS.

## Results

### Preliminary data analysis and selection of regulated features in PBMCs and plasma from ALS phenotypes

Quantitative analysis of all peptides and proteins was performed following normalisation within and between TMT 10plexes and values are expressed as log2 ratios relative to a common reference standard for each matrix (PBMCs, plasma). For selection of differentially expressed features, we excluded all peptides that were not detectable in all individual samples. In the PBMC experiment, the final data matrix comprised 2,757 distinct peptides representing 660 proteins, while in the plasma/brain discovery experiment, 4,905 distinct peptides and 1,126 proteins were quantified across all samples. To identify statistically significant regulated features, a modified t-test was performed, at the peptide and protein levels. Given the unbiased nature of the proteomics study and the need to maximise the identification of biomarker candidates within each study but also to increase the chance to find regulated proteins shared by plasma, brain and PBMCs, we opted for relatively relaxed thresholds for fold-change (+/− 30%) and significance (p ≤ 0.05) between classes.

Quantifiable outputs at peptide and protein levels are reported in Table 1. Individuals with different disease onset exhibit distinctive proteomic profiles in PBMCs and differences are more pronounced than in individuals with different progression rates, as demonstrated by the number of differentially-expressed peptides and proteins within the comparisons B-ALS vs L-ALS, B-ALS fast vs B-ALS slow and L-ALS fast vs L-ALS slow. The highest number of significantly regulated features in PBMCs was obseverved when fast B-ALS were compared to fast L-ALS (n = 1,039 peptides and n = 150 proteins). Based on this observation, a balanced number of B-ALS and L-ALS partecipants mostly with a fast progressing disease were selected for the plasma/brain proteomic study.

**Table 1:** Number of significantly regulated peptides and proteins based on a +/− 30% fold-change and p ≤ 0.05 comparing all disease phenotypes.

	Differentially-expressed peptides	Differentially-expressed proteins
**PBMC study**		
B-ALS vs L-ALS	1,003	132
B-ALS fast vs B-ALS slow	230	28
L-ALS fast vs L-ALS slow	185	20
B-ALS fast vs L-ALS fast	1,039	150
B-ALS slow vs L-ALS slow	502	88
**Plasma/brain study**		
B-ALS vs L-ALS	2,759	554

In the PBMC study, the comparison B-ALS versus L-ALS within the group of fast progressing patients showed the highest number of significantly regulated proteins and peptides. For this reason, for the plasma/brain proteomic study, bulbar and limb onset patients with a more rapid disease progression were selected.

### Evaluation of B-ALS vs L-ALS using principal component analysis

Following data normalisation and batch-effect removal, principal component analysis (PCA) on peptide and protein levels was performed using all quantified features from each matrix (PBMCs, plasma) irrespective of significance of regulation. The aim was to understand the influence of technical (TMT plex) and biological factors and guide subsequent data treatment for selection of significantly regulated features. For simplicity, we report here the outcomes from peptide level analysis only.

**Figure 1:**
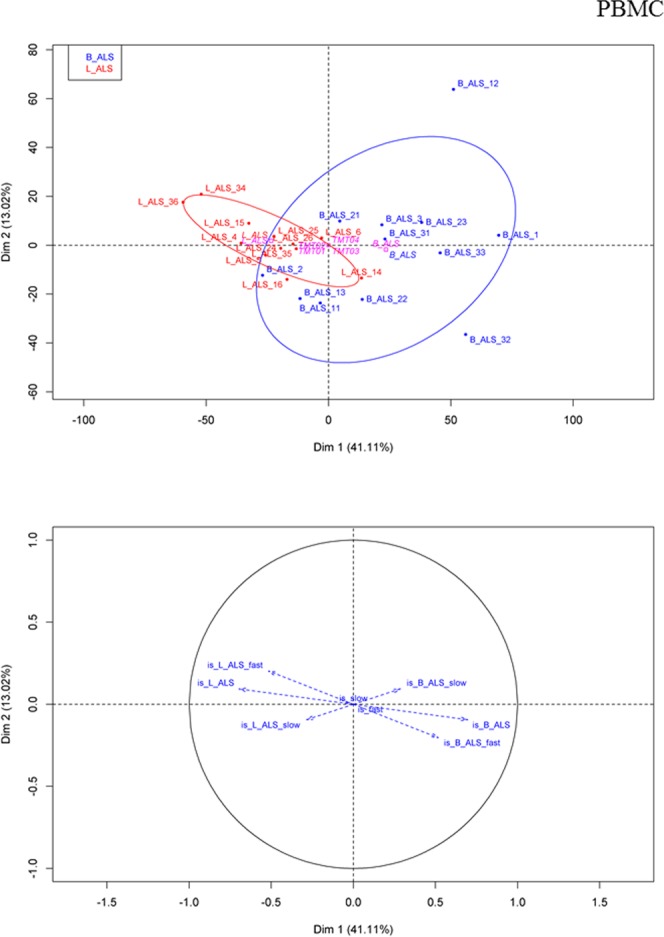
PBMC matrix - peptide level analysis. PCA score plot (upper) and loading plot (bottom) after median normalisation. In the PCA plot, B-ALS (blue) displays a higher degree of variability in the peptide profile compared to the L-ALS (red). The plot also shows a separation of the two clusters along the PC1 (x-axis) which is represented mostly by the site of disease onset as illustrated by the arrows in the loading plot. The loading plot confirms an anticorrelation between the B-ALS group and the L-ALS group, as indicated by the opposite direction of the corresponding vectors. A strong anticorrelation was also observed between B-ALS fast and L-ALS fast, as suggested by the lenght of the vectors.

In the PBMC matrix, the first principal component (PC1) accounted for 41.11% of the total data variance whilst the second principal component (PC2) accounted for 13.02% (Fig. 1).

The associated loading plot (Fig. 1 lower panel) shows a strong anticorrelation of B-ALS and L-ALS classes along PC1 and this is shown by the the opposite directions of the corresponding vectors and by their values (~−0.6–0.6) along the PC1. The rate of progression contributes to the separation along the PC2; however, the variance within the PBMC dataset is mostly driven by the site of disease onset.

**Figure 2:**
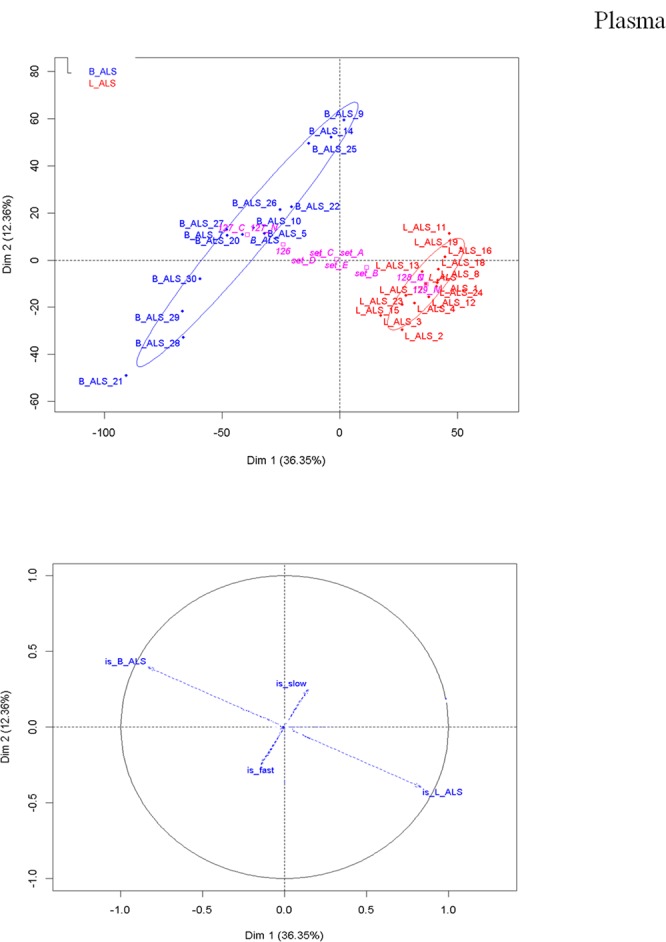
Plasma/brain matrix - peptide level analysis. PCA score plot (upper) and loading plot (bottom) after median normalisation. In the PCA plot, B-ALS (blue) displays a higher degree of variability in the peptide profile compared to the L-ALS (red) and this is in agreement with the results of the PBMCs. The loading plot shows that B-ALS group anticorrelates with the L-ALS group, as indicated by the opposite direction of the vectors representative of B-ALS and L-ALS. As observed in PBMC proteome, the rate of progression contributes to the separation along the PC2.

In the plasma/brain matrix, PCA after batch effect removal showed a similar, though weaker degree of separation between B-ALS and L-ALS patients, with PC1 accounting for 36.35% of the data variance (Fig. 2). As in the PBMC data, the B-ALS cloud is more disperse, suggesting a greater heterogeneity in patients with initial bulbar presentation. PC2 is mostly represented by the disease progression rate with PC2 accounting for 12.36% of variance, in agreement with the results from the PBMC proteome. B-ALS 21 appeared as an outlier, possibly because of the medical history of an autoimmune thyroid disorder while the remaining subjects under investigation presented no relevant co-morbidities in their medical history.

### PCA analysis using significantly regulated features

**Figure 3:**
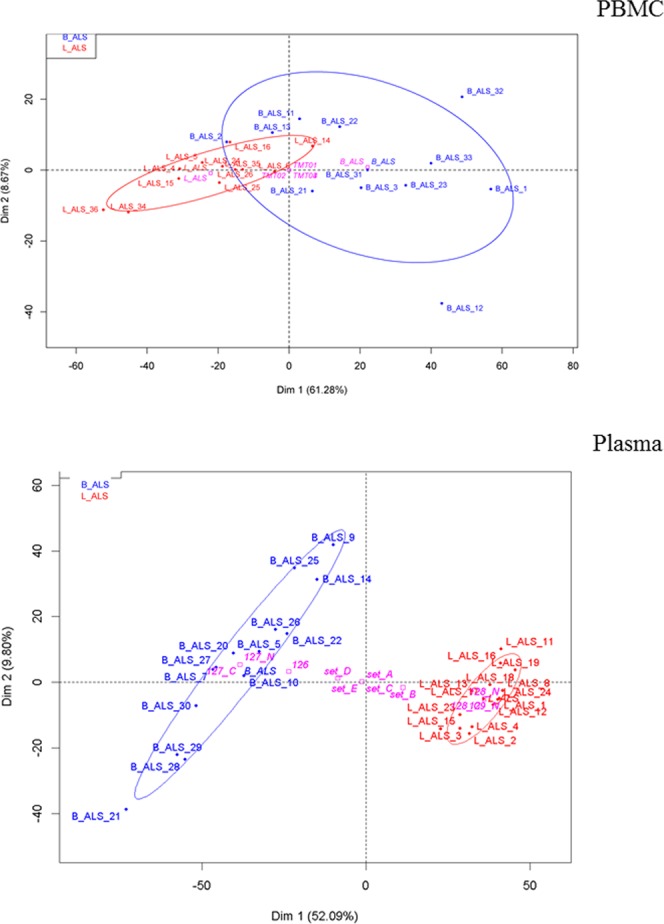
PBMC matrix (upper panel) and plasma/brain matrix (bottom panel) - peptide level analysis. PCA score plots of peptide data after feature selection shows a separation of the data mainly on PC1 in both matrices. Categorical factors such as TMT plex are reported in pink. B-ALS are shown in blue, L-ALS in red.

PCA plots were also generated using subsets of significantly differentially expressed features (p ≤ 0.05) extracted from the proteomic datasets. Results on the peptide level are provided in Fig. 3. In the PBMC matrix, the separation between B-ALS and L-ALS was stronger at peptide (Fig. 3 upper panel) and protein levels (data not showed) compared to the PCA score plot of the overall datasets after LIMMA-based batch effect removal (Fig. 1). The strength of separation in PC2 was reduced from 13.02% to 8.67%.

In agreement with the PBMC results, PCA of the significantly expressed features in the plasma proteome on a peptide level (Fig. 3 bottom panel) showed a better separation of the groups, compared to the plot after LIMMA-based batch effect removal (Fig. 2). The separation between B-ALS and L-ALS along the PC1 increased from 36.35% to 52.09% with the B-ALS cluster showing higher variability as the cloud appeared more disperse (Fig. 3 bottom panel). Also consistent with the PBMC analysis, the effect of separation on PC2 was slightly decreased from 12.36% to 9.80%.

These results show that the selected regulated features contribute significantly to the differences between B-ALS and L-ALS in both PBMCs and plasma/brain datasets. They also confirm that these features allow a better separation between phenotypes based on site of disease onset than to phenotypes with different progression rate.

Among the regulated features in both the two proteomic experiments, we found peptides mapping to myosin-9, fructose-bisphosphate aldolase A, heterogeneous nuclear ribonucleoproteins C1/C2 and isoform 7 of plectin (Fig. S4 Supplemental Data).

### Functional analysis of regulated features

Functional enrichment analysis using regulated peptide or protein features (|FC| > 1.3) was performed against a random selection of non-regulated features within the same data set. In addition, we have compared the regulated pathways in the PBMCs and plasma matrices to identify the presence of shared pathways when B-ALS is compared to L-ALS.

**Figure 4:**
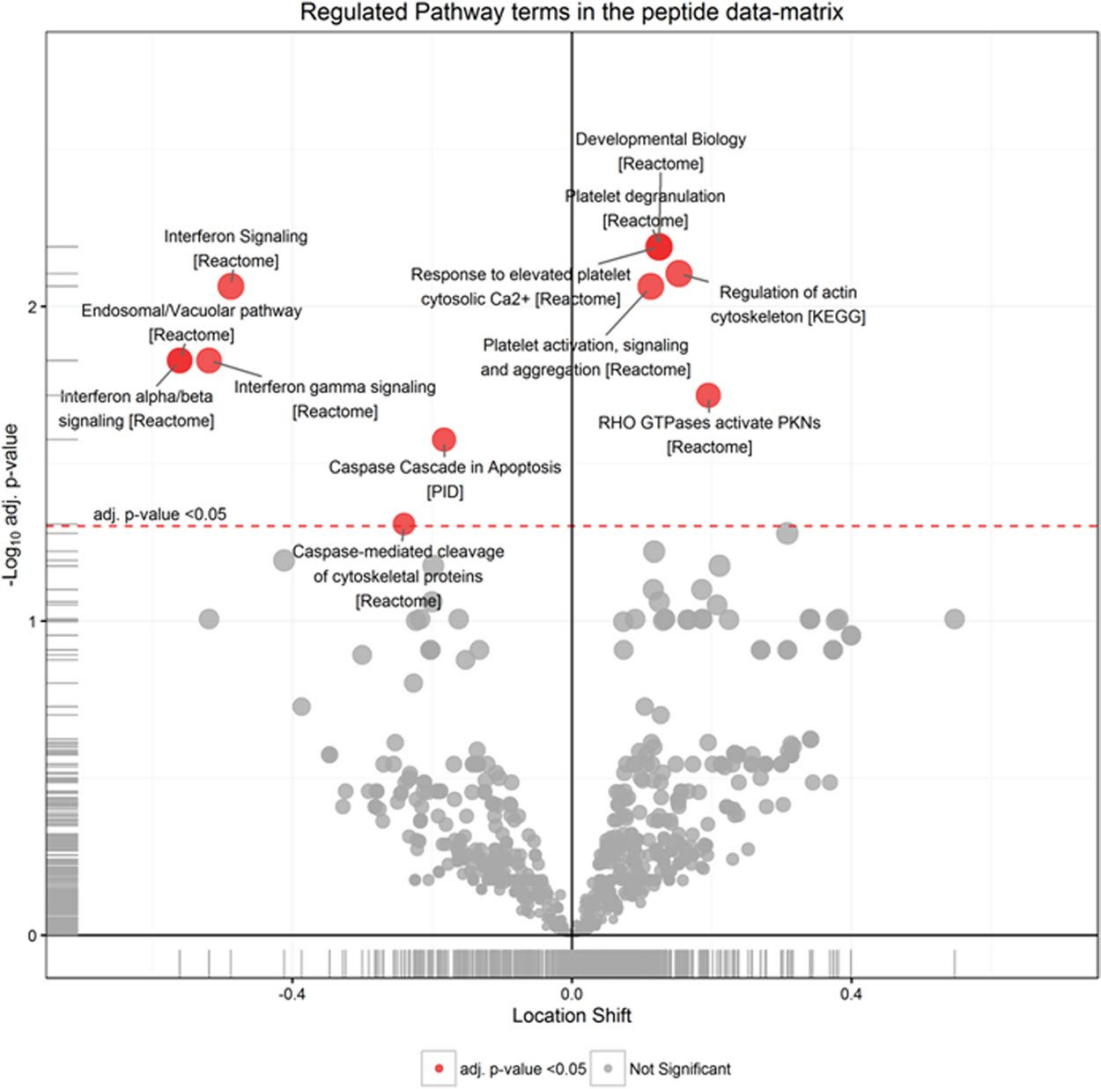
Significantly regulated pathways comparing B-ALS versus L-ALS at peptide level in PBMCs. Volcano plot showing medianFC against significance of enrichment (−log10 adj p-value), highlighting the pathways found to be significantly over- (right side of zero) or under- (left side of zero) represented by the differentially-expressed peptides and proteins in the data set. Filled red circles highlight significant pathway hits (adj p-value < 0.05).

Functional analysis of the regulated features in PBMCs from B-ALS versus L-ALS (Fig. 4 and Table 2) identified a significant enrichment of RHO GTPases, actin cytoskeleton, platelet activation/degranulation pathways, whereas pathways related to interferon, endosomal/vacuolar processes and caspase signaling were under-represented.

**Table 2:** Top20 regulated pathways mapped by regulated peptides identified in PBMC proteomes comparing B-ALS versus L-ALS (peptide level). Terms are sorted by adjusted p-values.

PBMCs_Top20 regulated Pathways	Effect size	adj. p-value
Developmental Biology	0.12	6.5E-03
Platelet degranulation	0.12	6.5E-03
Response to elevated platelet cytosolic Ca2+	0.12	6.5E-03
Regulation of actin cytoskeleton	0.15	7.9E-03
Interferon Signaling	−0.49	8.6E-03
Platelet activation, signaling and aggregation	0.11	8.6E-03
Endosomal/Vacuolar pathway	−0.56	1.5E-02
Interferon alpha/beta signaling	−0.56	1.5E-02
Interferon gamma signaling	−0.52	1.5E-02
RHO GTPases activate PKNs	0.19	1.9E-02
Caspase Cascade in Apoptosis	−0.18	2.7E-02
Caspase-mediated cleavage of cytoskeletal proteins	−0.24	4.9E-02
Systemic lupus erythematosus	0.31	5.3E-02
Signaling by Rho GTPases	0.12	6.0E-02
Immunoregulatory interactions between a Lymphoid and a non-Lymphoid cell	−0.41	6.4E-02
Nef-mediates down modulation of cell surface receptors by recruiting them to clathrin adapters	−0.70	6.7E-02
Nef mediated downregulation of MHC class I complex cell surface expression	−0.70	6.7E-02
Striated Muscle Contraction	−0.20	6.7E-02
EPHA-mediated growth cone collapse	0.21	6.7E-02
RHO GTPase Effectors	0.12	7.9E-02

**Figure 5:**
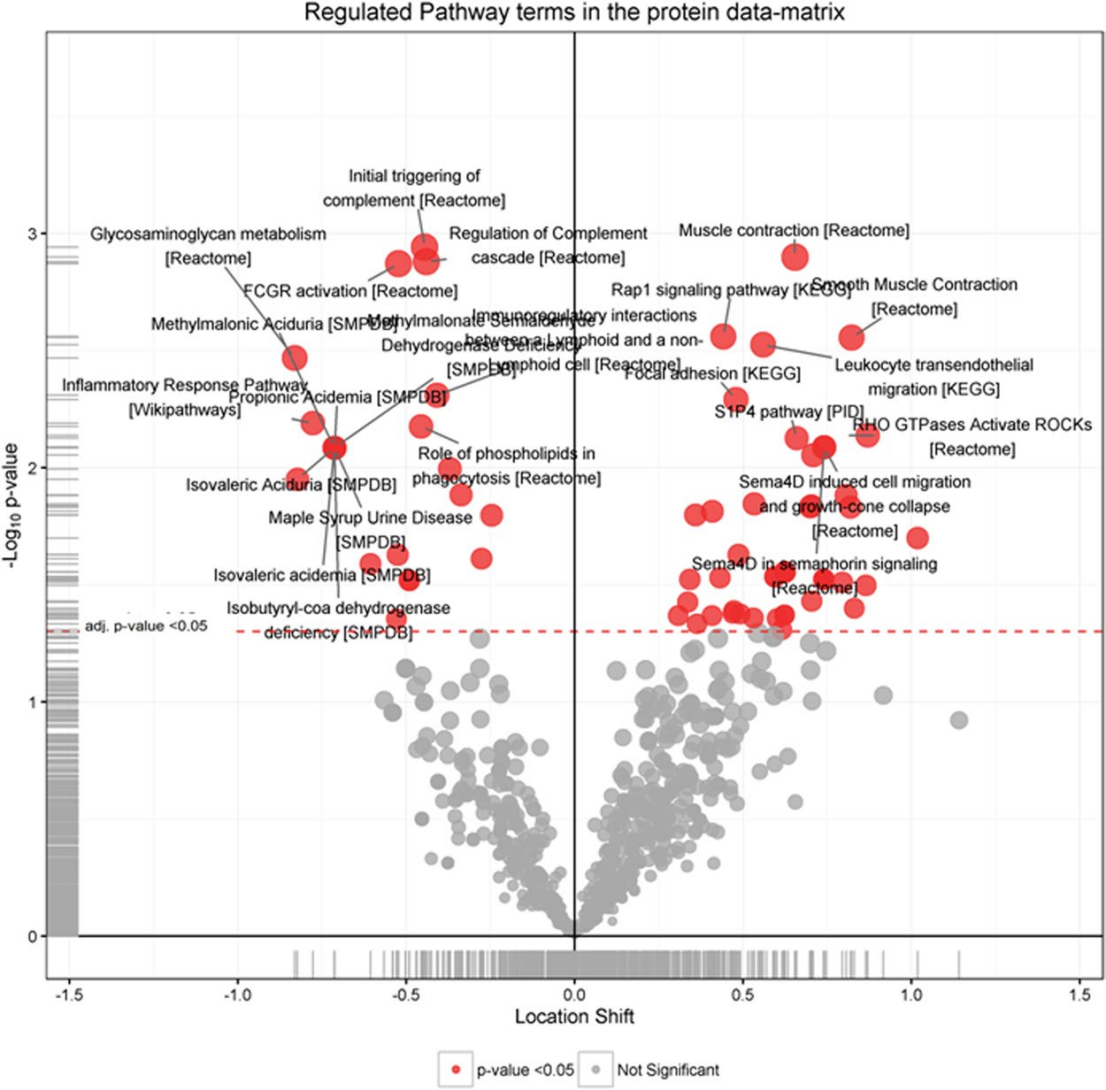
Significantly regulated pathways comparing B-ALS versus L-ALS at peptide level in plasma/brain. Volcano plot showing medianFC against significance of enrichment (-log10 adj p-value), highlighting the pathways found to be significantly over- (right side of zero) or under- (left side of zero) represented by the differentially-expressed peptides and proteins in the data set. Filled red circles highlight significant pathway hits (adj p-values = p-values and <0.05).

In the plasma/brain experiment, upregulated pathways in B-ALS versus L-ALS (Fig. 5 and Table 3) belonged to muscle contraction, smooth muscle contraction and leukocyte transendothelial migration. In this experiment, downregulated pathways were represented by initial triggering of complement, regulation of complement cascade, Fc gamma receptor (FCGR) activation and inflammatory response pathways.

**Table 3:** Top20 regulated pathways mapped by regulated peptides identified in plasma/brain proteomes comparing B-ALS versus L-ALS (peptide level). Terms are sorted by adjusted p-values.

Plasma_Top20 regulated Pathways	Effect size	adj.pvalue
Initial triggering of complement	−0.45	1.1E-03
Muscle contraction [Reactome]	0.65	1.3E-03
Regulation of Complement cascade	−0.44	1.3E-03
FCGR activation	−0.52	1.3E-03
Rap1 signaling pathway	0.44	2.7E-03
Smooth Muscle Contraction	0.82	2.8E-03
Leukocyte transendothelial migration	0.56	3.0E-03
Glycosaminoglycan metabolism [Reactome]	−0.83	3.4E-03
Immunoregulatory interactions between a Lymphoid and a non-Lymphoid cell	−0.41	4.9E-03
Focal adhesion	0.48	5.1E-03
Inflammatory Response Pathway	−0.78	6.4E-03
Role of phospholipids in phagocytosis	−0.46	6.7E-03
RHO GTPases Activate ROCKs	0.87	7.3E-03
S1P4 pathway	0.66	7.5E-03
Sema4D in semaphorin signaling	0.74	8.2E-03
Sema4D induced cell migration and growth-cone collapse	0.74	8.2E-03
Isobutyryl-coa dehydrogenase deficiency	−0.71	8.2E-03
Isovaleric acidemia	−0.71	8.2E-03
Isovaleric Aciduria	−0.71	8.2E-03
Maple Syrup Urine Disease	−0.71	8.2E-03

The following pathways were among the top 20 regulated pathways in both PBMCs and plasma/brain proteomic studies: immunoregulatory interactions between a lymphoid and a non-lymphoid cell, regulation of actin cytoskeleton, RHO GTPases activation of CIT, RHO GTPases activation of PAKs, RHO GTPases activation of ROCKs, salmonella infection, Sema4D in semaphorin signalling, Sema4D induced cell migration and growth-cone collapse and semaphorin interactions (Table 4).

**Table 4:** Significantly regulated pathways (p-value < 0.05) shared between the two studies.

Regulated pathways found in PBMCs and plasma proteomes	Matched proteins in PBMCs	Matched proteins in Plasma
Immunoregulatory interactions between a Lymphoid and a non-Lymphoid cell	Immunoglobulin kappa constant, HLA class I histocompatibility antigen, A-2 alpha chain, Integrin beta-1	Tetraspanin, Complement C3, Immunoglobulin kappa variable 1–39, Immunoglobulin kappa variable 1–5, Immunoglobulin kappa variable 3–20, Immunoglobulin kappa variable 4–1, Immunoglobulin lambda variable 1–47, Immunoglobulin lambda variable 3–19, Immunoglobulin lambda variable 3–25, Immunoglobulin heavy variable 1–69, Immunoglobulin heavy variable 3–11, Immunoglobulin heavy variable 3–23, Immunoglobulin heavy variable 3–23, Immunoglobulin heavy variable 3–7, Immunoglobulin heavy variable 4–39, Immunoglobulin heavy variable 4–39, Collagen alpha-1(I) chain, Immunoglobulin kappa variable 3–15, Immunoglobulin lambda variable 1–47, Beta-2-microglobulin, Immunoglobulin lambda variable 3–21, Intercellular adhesion molecule 5
Regulation of actin cytoskeleton	Actin-related protein 2/3 complex subunit 1B, Actin-related protein 2/3 complex subunit 2, Actin-related protein 2/3 complex subunit 5, Alpha-actinin-4, Integrin beta-3, Integrin beta-1, Profilin-1, Integrin alpha-M, Ras-related C3 botulinum toxin substrate 2, Ezrin	Cytoplasmic FMR1-interacting protein 2, Actin-related protein 2/3 complex subunit 2, Actin-related protein 2/3 complex subunit 3, Alpha-actinin-4, Prothrombin, Gelsolin, Profilin-1, Proto-oncogene tyrosine-protein kinase Src, Myosin regulatory light chain 12A, Moesin, Mitogen-activated protein kinase 3, Mitogen-activated protein kinase 1, Radixin, Myosin-9, Alpha-actinin-2, Dual specificity mitogen-activated protein kinase kinase 2, Actin, cytoplasmic 1, Cell division control protein 42 homolog, Transforming protein RhoA, Serine/threonine-protein phosphatase PP1-alpha catalytic subunit, Serine/threonine-protein phosphatase PP1-beta catalytic subunit, Thymosin beta-4, Ras-related C3 botulinum toxin substrate 1, Phosphatidylinositol 5-phosphate 4-kinase type-2 beta, Serine/threonine-protein kinase PAK 1, Myosin-14, Actin-related protein 2/3 complex subunit 5-like protein, Nck-associated protein 1
RHO GTPases activate CIT	Myosin-9, Transforming protein RhoA	Myosin-9, Transforming protein RhoA, Rho-related GTP-binding protein RhoB, Myosin-14, Ras-related C3 botulinum toxin substrate 1
RHO GTPases activate PAKs	Myosin-9, Cell division control protein 42 homolog, Calmodulin-1, Calmodulin-2, Calmodulin-3, Serine/threonine-protein kinase PAK 2	Myosin-9, Cell division control protein 42 homolog, Serine/threonine-protein phosphatase PP1-beta catalytic subunit, Ras-related C3 botulinum toxin substrate 1, Serine/threonine-protein kinase PAK 1, Filamin-A, Myosin-14
RHO GTPases Activate ROCKs	Myosin-9, Transforming protein RhoA	Myosin-9, Transforming protein RhoA, Myosin-14, Serine/threonine-protein phosphatase PP1-beta catalytic subunit, Rho-related GTP-binding protein RhoB
Salmonella infection	Actin-related protein 2/3 complex subunit 1B, Actin-related protein 2/3 complex subunit 2, Actin-related protein 2/3 complex subunit 5, Profilin-1, Myosin-9, Actin-related protein 2/3 complex subunit 4, Actin, cytoplasmic 1, Cell division control protein 42 homolog, Rho-related GTP-binding protein RhoG	Actin-related protein 2/3 complex subunit 2, Actin-related protein 2/3 complex subunit 3, Profilin-1, Mitogen-activated protein kinase 3, Mitogen-activated protein kinase 1, Myosin-9, Ras-related protein Rab-7a, Actin, cytoplasmic 1, Cell division control protein 42 homolog, Ras-related C3 botulinum toxin substrate 1, Rho-related GTP-binding protein RhoG, Tight junction protein ZO-1, Cytoplasmic dynein 1 heavy chain 1, Fatty acid-binding protein, liver, Myosin-14, Actin-related protein 2/3 complex subunit 5-like protein
Sema4D in semaphorin signaling	Myosin-9, Transforming protein RhoA, Ras-related C3 botulinum toxin substrate 2, Cell division control protein 42 homolog, Rho-related GTP-binding protein RhoG	Myosin-9, Cell division control protein 42 homolog, Transforming protein RhoA, Rho-related GTP-binding protein RhoB, Ras-related C3 botulinum toxin substrate 1, Rho-related GTP-binding protein RhoG, Myosin-14
Sema4D induced cell migration and growth-cone collapse	Myosin-9, Transforming protein RhoA, Ras-related C3 botulinum toxin substrate 2, Cell division control protein 42 homolog, Rho-related GTP-binding protein RhoG	Myosin-9, Cell division control protein 42 homolog, Transforming protein RhoA, Rho-related GTP-binding protein RhoB, Ras-related C3 botulinum toxin substrate 1, Rho-related GTP-binding protein RhoG, Myosin-14
Semaphorin interactions	Integrin beta-1, Heat shock protein HSP 90-alpha, Heat shock protein HSP 90-beta, Ras-related C3 botulinum toxin substrate 2, Cofilin-1, Myosin-9, Cell division control protein 42 homolog, Transforming protein RhoA, Rho-related GTP-binding protein RhoG, Serine/threonine-protein kinase PAK 2, Talin-1	Dihydropyrimidinase-related protein 4, Heat shock protein HSP 90-alpha, Heat shock protein HSP 90-beta, Myosin-9, Cell division control protein 42 homolog, Transforming protein RhoA, Rho-related GTP-binding protein RhoB, Ras-related C3 botulinum toxin substrate 1, Rho-related GTP-binding protein RhoG, Cyclin-dependent-like kinase 5, Serine/threonine-protein kinase PAK 1, Dihydropyrimidinase-related protein 1, Dihydropyrimidinase-related protein 2, Myosin-14, Dihydropyrimidinase-related protein 5, Talin-1

Proteins whose peptides were found regulated in the two data sets are reported.

### ALS proteins quantified in PBMCs and plasma discovery studies

In our discovery studies we quantified proteins previously linked to the pathogenesis of ALS such as fibronectin (isoform 12–14), profilin-1 and superoxide dismutase. RNA-binding protein FUS and superoxide dismutase Cu-Zn were found significantly regulated (p-value < 0.01) in PBMC at protein level. Alpha-internexin, complement C3, neurofilament light polypeptide, neurofilament medium polypeptide, profilin-1, serum amyloid P-component and transthyretin were found significantly regulated (p-value < 0.01) in depleted plasma at protein level (Fig. S5 Supplemental Data).

### Immunodetection of neurofilaments light, neurofilaments heavy and apoE in plasma

To confirm that the expression and regulation of ALS-relevant proteins identified in our plasma proteomic study could be reproduced using an orthogonal technique of immunodetection, plasma levels of three well-known ALS proteins (Fig. S5 Supplemental Data) i.e. neurofilament light and heavy (NfL, NfH) polypeptides and apolipoprotein E (ApoE; Fig. S5 Supplemental Data) were tested.

In the plasma/brain discovery study comparing B-ALS to L-ALS, samples were obtained from ALS patients with a relatively fast progression of the disease (progression rate to last visit, PRL > 0.7). In the discovery study, NfL was significantly regulated in plasma from B-ALS compared to L-ALS (logFC = −0.49 p-value = 0.008), whereas NfH did not show any significant regulation between B-ALS and L-ALS (logFC = 0.16 p-value = 0.13). ApoE was detected as non-significantly regulated when comparing B-ALS to L-ALS (logFC = 0.0 p-value = 0.99).

**Figure 6:**
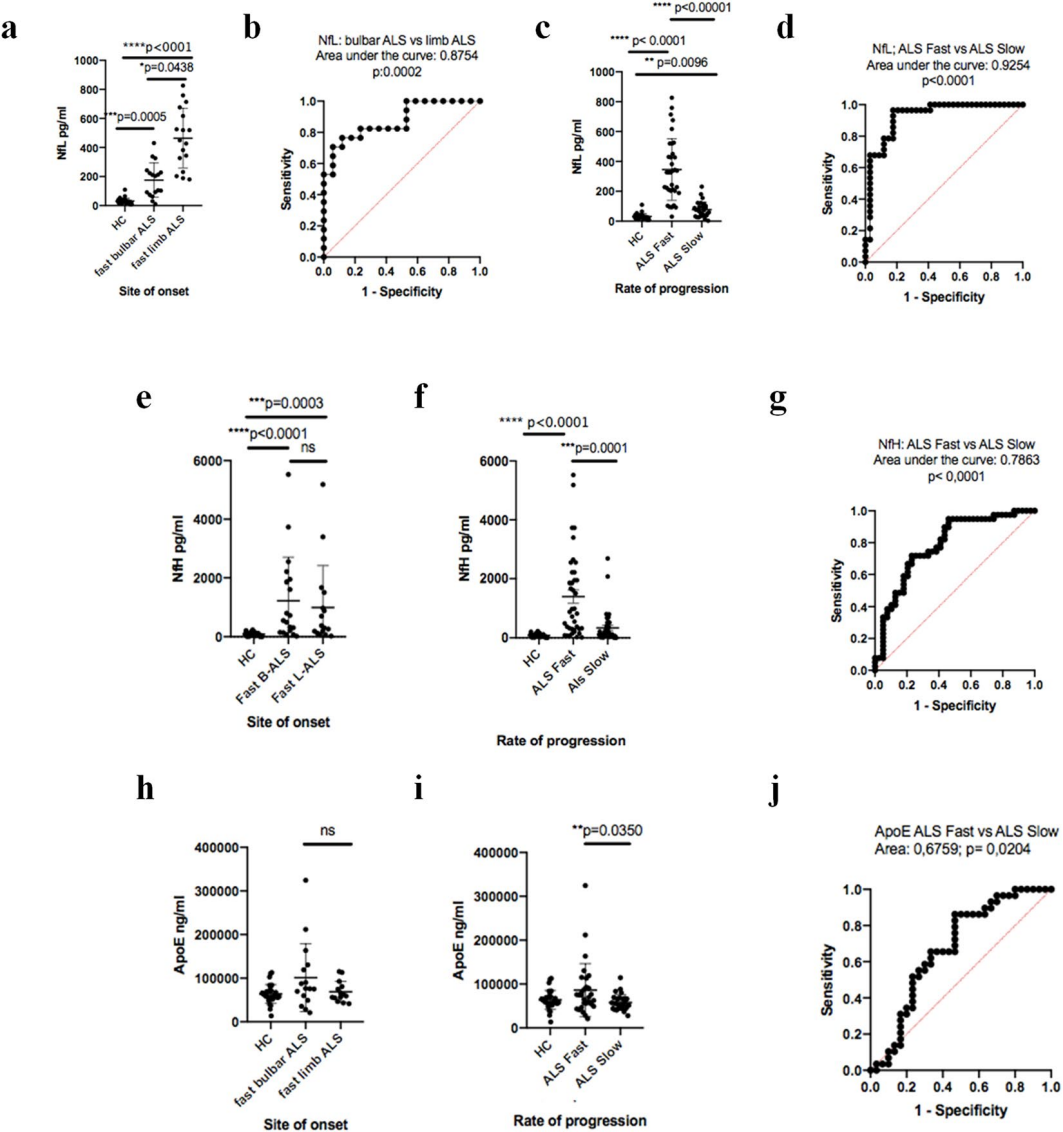
Single molecular array (Simoa) and Mesoscale discovery (MDS) immunodetection of plasma protein candidates selected from the PBMCs and brain/plasma proteomic studies. In a re-test cohort of B-ALS and L-ALS patients with a fast (PRL > 0.7) and slow (PRL < 0.7) progressing disease immunodetection results are in line with the findings of the proteomic experiments. (**a)** Neurofilament light polypeptide (NfL) is overexpressed in plasma from limb onset ALS patients with a relatively fast progressing disease compared to bulbar onset ALS patients with a similar rate of disease progression (p = 0.0438), whilst plasma NfL expression in both bulbar and limb onset fast progressing ALS patients is significantly up-regulated compared to healthy controls (p = 0.0005 and p < 0.0001, respectively). (**b)** Receiver operating characteristic (ROC) shows NfL good performance in separating fast B-ALS from fast L-ALS (area under the curve (AUC): 0.8754 p = 0.0002). (**c)** Plasma NfL in fast and slow progressing ALS patients is significantly different from healthy controls as previously reported^4^, whilst (**d)** plasma NfL ROC is highly performant in fast vs slow-progressing ALS patients separation (AUC: 0.9254 p < 0.0001). (**e)** Plasma neurofilament heavy polypeptide (NfH) in fast B-ALS patients has higher but not significantly different expression than fast L-ALS patients, while plasma NfH expression in both subgroups is significantly upregulated compared to healthy controls (p < 0.0001 and p = 0.0003, respectively). (**f**) Plasma NfH in fast progressing ALS (but not in ALS slow) is significantly higher compared to healthy controls. (**g**) ROC indicates that NfH separates well fast progressing bulbar vs limb onset disease (AUC: 0.7863 p < 0.0001). (**h**) Plasma ApoE expression is higher in fast B-ALS compared to fast L-ALS but not statistically significant, whilst plasma ApoE is moderately over-expressed in fast progressing ALS compared to slow progressing ALS (p = 0.0350) as previously reported^18^. (**j**) ROC analysis of ApoE expression in ALS-fast and ALS-slow shows a modest performance in the separation of the two sub-groups (AUC:0.6759 p = 0.024).

From a re-test cohort of 82 ALS patients for the immunodetection experiments, we have also selected B-ALS (n = 20) and L-ALS (n = 17) patients with a fast disease progression (PRL > 0.7) and healthy controls (n = 29). Group analysis of the immunodetection results in the three sub-groups, showed that NfL, NfH and ApoE had the same patterns of expression observed in the plasma/brain TMTcalibrator study (Fig. 6). Analysis of diagnostic performance using receiver operating characteristic (ROC) showed that NfL expression differentiated very well B-ALS from L-ALS and ALS-fast from ALS-slow, while NfH and ApoE expression provided only a discrete level of differentiation between ALS-fast and ALS-slow.

## Discussion

In this study, we used two complementary TMT proteomic workflows to identify biological signals in easily accessible PBMCs and plasma samples from clinically homogeneous sub-sets of ALS patients representing phenotypic extremes of ALS. We have characterized peptides expressed in PBMCs, plasma and brain that undergo regulation in specific phenotypic variants of the disease, more significantly in B-ALS compared to L-ALS in a fast progressing disease state. These findings provide a strong ground to develop biomarkers for clinical stratification in ALS. Prior to enrolment in clinical trials, patient stratification using biological readouts can be used to mitigate phenotypic heterogeneity which is a major confounder and possibly one of the main reasons for the failure of over 50 ALS clinical trials in the last 20 years^19^. Bulbar onset disease and signs of fronto-temporal involvement are not the only features linked to a worse prognosis in ALS. The speed of functional decline and the overall survival appear also to depend on the presence of other factors including older age at onset, female gender, lower body max index, shorter latency from symptom onset to diagnosis, lower initial ALS functional rating scale revised (ALSFRS-R) and non-use of Riluzole^3^, the first and until recently the only drug licenced to treat ALS. From a genetic and biochemical standpoint, the UNC13A (rs12608932 minor allele), lower uric acid, serum creatinine and albumin levels are also reported in association with a more severe disease phenotype^20,21^.

PCA analysis of the PBMCs and plasma/brain proteomes from the phenotypic variants under investigation shows that the site of disease onset is the main contributor to the overall biological differences across the chosen phenotypes. The B-ALS cluster in both PCA plots appears more disperse than the one seen in L-ALS, suggesting a higher degree of variability in the bulbar proteomic profile in PBMCs and plasma compared to the limb profile. Moreover, the bulbar versus limb differential protein expression is more pronounced in the sub-population of ALS patients with the highest rate of disease progression and this finding is strengthened by the study of three ALS-relevant proteins which have been tested by immundetection in a much larger cohort of fast progressing (bulbal and limb onset) ALS individuals. These observations are difficult to explain based on current knowledge on the pathobiology of the disease. From a clinical perspective, bulbar and limb onset ALS diverge significantly over the course of the disease, with a different patters of clinical progression which include the pathological involvement of cortical function with features of fronto-temporal dementia possibly more prevalent in bulbar-onset and fast progressing ALS patients^1,2^. This study looks at changes of the proteomic profile in both the immunological cells of the peripheral blood, plasma and in the brain tissue, showing how in such distant biological substrates, it is still possible to dissect the molecular signature of bulbar onset disease and separate this from the limb-onset pathology. Such a phenotype-specific molecular footprint may suggest that treatments may only be effective if directed against the disease when the pathology can be pinned down to a defined molecular profile, supporting the long-held view that clinical trials have to be conceived with as homogeneous as possible patient groups.

The choice of ALS cases to be included in the two proteomic discovery experiments and in the larger re-test cohort was dictated by the need of applying very stringent criteria for the selection of phenotypically homogeneous subsets of ALS patients and by the goal of obtaining reproducible data across biological systems, in different subsets of neurological patients stratified according to the same clinical parameters. The identification in the PBMC study of a larger number of protein candidates expressed and regulated in fast progressing ALS patients with bulbar onset has dictated the selection of a separate larger cohort of fast progressing ALS patients for the second stage discovery study, the plasma/brain TMTcalibrator. This strategy has generated a comparable pattern of regulated pathways across the two proteomic studies. The expression and regulation of candidates already known to be relevant to ALS pathology, including neurofilaments and ApoE, has then been re-tested and confirmed using immunodetection in a separate cohort of ALS individuals which reproduce the same phenotypic characteristics of the subsets of patients included in the discovery experiments.

The high sensitivity and the multiplex capacity of the two TMT-MS platforms employed in this study have allowed the identification of significantly regulated molecules across several samples in the clinical phenotypes under investigation, even though the TMT-MS2 discovery approach applied to the analysis of PBMC proteome was based on a more straighforward sample preparation (absence of SCX fractionation) than the TMTcalibrator. In the TMTcalibrator, we have obtained a deep coverage of the ALS plasma proteome using (i) ALS brain as a trigger of ALS-associated proteins and (ii) SCX fractionation before the MS analysis. Simultaneous in-depth proteomic analysis of plasma samples allowed the detection of both highly abundant plasma proteins (such as apolipoprotein B, ceruloplasmin, complement C3, plasminogen, etc) and of numerous lower abundance neuronal and glial proteins (brain acidic soluble protein 1, neuronal cell adhesion molecule 1, gamma-aminobutyric acid receptor-associated protein-like 2, neurogranin, glial fibrillary acidic protein, isoform 3 of myelin basic protein and aquaporin 4). The two experiments have shown some degree of intersection but also a matrix related specificity on the protein profiles identified, while both approaches have been able to differentiate B-ALS from L-ALS with a good degree of separation. The presence of regulated neuronal and glial proteins in plasma samples might suggest a blood-brain (BBB) and/or blood-spinal cord barrier (BSCB) perturbation which was previously reported in ALS^22–24^. We cannot infer whether the regulation of the proteins identified in this study are a causal factor for BBB/BSCB damage or their presence in plasma is a consequence of the reported barrier alterations.

We have found regulated proteins already known to be under investigation as biomarkers of neurodegeneration and other whose biological significance in neurodegeneration and in the context of ALS may still be unexplored. Our workflows allowed the detection of several peptides mapping to well-known ALS proteins (Fig. S5 Supplemental Data) and the altered protein levels were not related to the presence of any mutations as all our patients were sporadic cases with no evidence of known ALS genetic mutations. This outcome somehow strengthens the reliability of this approach and its potential use for exploratory studies providing reliable molecular information on different ALS phenotypes. For example, glial fibrillary acidic protein (GFAP) was previously reported in blood and its increased levels were found in patients with different neurological diseases, as a possible consequence of astroglial destruction^25^. Additionally, we had evidence of peptides mapping neurofilament medium polypeptide (NfM), an axonal protein which was previously detected in blood using commercially available ELISA kits and found up-regulated in CSF and serum from patients with brain injury^26^. We have also identified NfL and NfH and re-tested them using immunodetection along-side ApoE. These proteins have been already extensively investigated by us and other groups and bear potential as biomarkers of disease stratification in ALS^4,18^.

The assessment of the shared regulated pathways emerging from the functional analysis performed in the PBMCs and plasma datasets when comparing B-ALS versus L-ALS showed a significant regulation of immunoregulatory interactions between lymphoid and non-lymphoid cells, RHO GTPases and Sema4D signalling. Immune mediators linked to B and non-B lymphocyte activation are extensively investigated in ALS for the purpose of establishing a way to immunomonitor the disease^27^. RHO GTPases have a regulatory role in actin and microtubule cytoskeletons formation, gene transcription, cell–cell adhesion, cell cycle progression, neuronal apoptosis and modulation of microglial activity^28,29^. Within pathways involved in the regulation of the inflammatory response, actin cytoskeleton and in Sema4D signalling for cell migration and growth-cone collapse, we have identified differentially expressed proteins which are central to cell-cell and cell-extracellular matrix cross-talk. Among these, integrin beta-1, beta-3 and alpha-M are certainly a significant hit in our discovery study, in virtue of their functional involvement in cell adhesion and angiogenesis and for their reported characteristic brain expression in microglia (beta-3, alpha-M) and in endothelial cells (beta-1), according to a comprehensive RNA-Seq transcriptome and splicing database of glia, neurons, and vascular cells of the cerebral cortex (https://web.stanford.edu/group/barres_lab/brain_rnaseq.html). Integrins and vascular endothelial growth factor (VEGF), a neuroprotective hypoxia-related regulator of angiogenesis strongly linked to the pathogenesis of ALS, are currently the target of therapeutic strategies aimed at modifying angiogenesis in both neurodegeneration and in malignat form of metastatic cancer^30,31^. A physiological role in promoting angiogenesis and neuronal survival has also emerged for angiogenin following the discovery of genetic mutations of the gene encoding for this protein in familial forms of ALS and Parkinson’s disease^32^. Emphasis on the potential role of integrins in disease modification and monitoring in ALS comes also from the observation that position electron tomography imaging and radioligands targeting different integrin isoforms have been developed for the monitoring of specific forms of cancer, an approach that could also be suitable as diagnostic biomarker in ALS^33^. The significant differential regulation of a semaphoring signaling pathway (Sema4D), involved in neurite outgrouth and angiogenesis, adds to previous experimental observatios highlighting the role of these factors in neurological conditions like MS as well as in neuronal injury and repair via T-cells signalling modification^34,35^.

In conclusion, the data sets generated from our protemic workflows in combination with our in-house bioinformatic pipeline has dissected a range of peripheral molecular changes which could be further investigated as early diagnostic tests to differentiate clinical phenotypes of ALS. While validation studies are needed to establish the specificity and clinical utility of relevant sets of proteins in larger cross-sectional and longitudinal cohorts, this investigation provides a range of molecular signals that may have not been otherwise detectable in a simple healthy versus disease experimental setting, widening the spectrum of relevant biological features that could be exploited for biomarker verification and for novel therapeutic strategies.

## Methods

### Patient selection

#### PBMC study cohort

Ethical approval was obtained from the East London and the City Research Ethics Committee 1 (09/H0703/27) for the inclusion of all ALS patients participating in both the PBMCs and in the plasma/brain study. Informed consent was obtained from all participants. All research was performed in accordance with relevant guidelines/regulations.

Twelve bulbar-onset ALS patients (B-ALS) with no clinical and neurophysiological evidence of more widespread disease for at least twelve months following diagnosis and twelve limb-onset ALS patients (L-ALS) with a diagnosis of probable, laboratory-supported probable or definite ALS (El-Escorial criteria) were recruited from the ALS clinics (Table 5). The level of neurological impairment was assessed using the ALS Functional Rating Scale revised (ALSFRS-R)^36^, which allows rating of different domains of disability (score range: 1–48, increasing disability with lower scores). Disease progression was calculated using the monthly ALSFRS-R slope throughout the clinical follow-up to the last visit (progression rate to last visit (PRL) = 48 minus ALSFRS-R score at last visit/duration of disease from symptoms onset to last visit in months). An equal proportion of fast and slow progressing ALS cases were selected (i.e. 6 subjects with a PRL < 0.5 and 6 subjects with a PRL > 1.5 within each B-and L ALS group). N = 7 B-ALS and n = 7 L-ALS patients were taking riluzole at the time of sampling.

**Table 5:** PBCMs study cohort demographics.

	PRL		Gender			Ethnic group		Age of onset (year range)	Diagnostic latency (month range)	ALSFRS-R (/48) at blood withdraw (score range)	Cognitive impaired patients	Riluzole
	<0.5 slow	>1.5 fast	female	male	NA	caucasian	asian					
**B-ALS (n = 12)**												
	6	6	6	5	1	10	2	57–83	3–28	27–45	3	7
**L-ALS (n = 12)**												
	6	6	2	10		11	1	32–70	2–74	24–48	1	7

Legend: PRL: progression rate to last visit. ALSFRS-R: Revised ALS Functional Rating Scale.

#### Plasma/brain study cohort

In consideration of the fact that the highest number of significantly regulated features in the PBMC study were found comparing B-ALS vs L-ALS samples in the fast progressing ALS subgroup (Table 1), a larger number of fast progressing ALS individuals were selected in the subsequent plasma/brain TMTcalibrator study to identify proteins expressed in plasma that may be co-expressed in PBMCs and related to a particularly aggressive form of the disease.

Fourteen B-ALS cases and sixteen L-ALS cases were recruited from the ALS clinics (Table 6). Neurological impairment and PRL were assessed as reported above. Proteomic analysis was performed on samples obtained from four subjects with a PRL < 0.4 (slow progressors) and twenty-five subjects with a PRL > 0.7 (fast progressors) were selected. N = 11 B-ALS and n = 13 L-ALS patients were taking riluzole. Given the highly specific selection criteria in the study which include with bulbar onset ALS with no signs of more widespread disease for at least 12 months from diagnosis, as well as the population of available patients and the relative rarity of the chosen phenotypes, a less stringent PRL cut-off was chosen.

**Table 6:** Plasma study cohort demographics.

	PRL		Gender			Ethnic group		Age of onset (year range)	Diagnostic latency (month range)	ALSRS-R (/48) at blood withdraw (score range)	Cognitive impaired patients	Riluzole
	<0.4 slow	>0.7 fast	female	male	NA	caucasian	asian					
**B-ALS (n = 14)**												
	2	12	4	10		12	2	45–85	1–12	8–46	5	11
**L-ALS (n = 16)**												
	2	14	5	11		14	1	40–76	2–24	21–47	3	13

Legend: PRL: progression rate to last visit. ALSFRS-R: Revised ALS Functional Rating Scale.

For all patients included in the study, C-reactive protein levels were found to be <2 mg/L in blood samples from which PBMCs and plasma were separated.

Brain tissue (pre-central girus cortex) was obtained from the Dutch National Brain Tissue Bank under ethical approval.

### Re-test cohort

Plasma samples from a total of thirtyeight B-ALS and fourty-four L-ALS patients were identified to re-test by immunodetection the expression of selected biomarkers which have emerged in the PBMC and plasma/brain proteomic study. Twentynine healthy individuals were also included (Table 7). For the re-test cohort, site of disease onset was the most prominent functional impairment observed at the outset, including dysarthria (bulbar) or limb weakness (limb). However, unlike the discovery cohorts used for the proteomic studies, in most ALS individuals in this subset, the initial prominent clinical features were either associated or shortly followed by neurological and neurophysiological signs of more widespread disease. The ALS patients were also categorized according to rate of disease progression in ALS fast (PRL > 0.7) and ALS slow (PRL < 0.4). None of the selected ALS individuals and healthy controls had a previous history of neurotrauma or brain and systemic inflammtory/metabolic disorder.

**Table 7:** Clinical and demographic characteristics of the cohort of ALS individuals and healthy controls (HC) selected for the re-test experiment of neurofilament light (NfL), heavy (NfH) polypeptides and of apolipoprotein E (ApoE) using immunodetection.

	PRL		Gender		Ethnic group		Age of onset (year range)	Diagnostic latency (month range)	ALSRS-R (/48) at sampling (score range)	PRL (range)
	<0.4 slow	>0.7fast	female	male	caucasian	asian				
**B-ALS (n = 38)**										
	12	26	17	21	37	1	39–76	1.3–71.3	13–44	0.3–1.8
**L-ALS (n = 44)**										
	27	17	19	25	44	—	33–81	2–129.9	23–47	0.1–2.4
							**Age at sampling**			
**HC (n = 29)**										
	NA		19	10	29	—	50–73			NA

In the plasma/brain proteomic experiment, ALS patients were sub-grouped according to rate of disease progression, where fast progressing individuals had a progression rate to last visit (PRL) >0.7 and slow progressing individuals had a PRL < 0.4. To study the selected biomarkers expression in fast progressing disease (as in the plasma/brain proteomic experiment), only bulbar and limb-onset ALS individuals (B-ALS and L-ALS) with a PRL > 0.7 were selected for immunodetection analysis.

### Sample preparation

#### PBMC processing

Whole blood was collected via venepuncture into 6 mL EDTA tubes (BD Vacutainer). Samples were processed within 1 h from blood collection. PBMCs were isolated from blood by density-gradient centrifugation at 1,500 g for 40 min (20 °C) using Lymphoprep™ (Alere) and washed in Dulbecco’s phosphate-buffered saline (Gibco). PBMC pellets were stored at −80 °C in 10% DMSO and foetal bovine serum. After 24 hours, PBMCs were transferred to liquid nitrogen.

### PBMC lysis

Prior to proteomic analysis, PBMCs were thawed by placing cryovials in a 37 °C water bath for 10 min and washed as previously reported^37^. After gentle centrifugation at 330 g for 10 min, surnatants were removed and PBMC pellets were suspended in 8 M Urea, 75 mM sodium chloride (NaCl), 50 mM Tris buffer pH 8.2 and protease and phosphatase inhibitors (Roche), vortexed for 5 min and sonicated for 10 sec three times on ice. Samples were centrifuged at 14,000 g for 10 min at 4 °C. Supernatants were aliquoted and stored at −80 °C. Protein concentration was assessed by Bradford assay (Fig. S1 and Table S1 Supplemental Data).

### Digestion, TMT labeling and clean-up

20 µg of protein extract was diluted to 1 µg/µL by adding 100 mM of triethylammonium bicarbonate (TEAB) buffer containing 0.1% sodium dodecyl sulphate (SDS). Proteins were reduced for 60 min at 55 °C by the addition of 5.3 µL of 20 mM aqueous tris(2-carboxyethyl)phosphine (TCEP) and alkylated for 60 min at room temperature by the addition of 5.5 µL of 150 mM iodoacetamide in ACN. 10 µL of 0.4 µg/µL trypsin (Promega) in 100 mM TEAB was added and the sample was incubated at 37 °C for 18 h. Digested proteins were labelled by adding 40.3 µL of a 60 mM TMT10 reagents (Thermo Scientific) in acetonitrile (ACN) for 1 h at room temperature. 8 µL of an aqueous hydroxylamine solution (5%, w/v) was added and incubated for 15 min at room temperature. All 10 TMT-labeled samples were mixed together to generate four TMT 10plexes. Each TMT 10plex was desalted using vacuum pump and Oasis HLB 1 cc 30 mg extraction cartridges (Waters) according to manufacturer’s instruction then lyophilized. Samples were solubilised in 5% ACN and 0.1% trifluoroacetic acid (TFA) and then desalted. Following lyophilisation, all vials were stored at −80 °C. Fig. S2 illustrates the experimental study design.

#### Plasma and brain processing

Plasma samples from the discovery study and re-test were extracted from whole blood collected into EDTA tubes (BD Vacutainer). All samples were processed within 1 h from blood collection. Following centrifugation at 3500 rpm 10 min at 20 °C plasma samples were aliquoted and frozen at −80 °C (0.5 mL/aliquot in polypropylene tubes with screw caps).

The plasma samples processed for the discovery TMTcalibrator study were depleted of albumin and IgG (ProteoPrep Immunoaffinity Albumin and IgG Depletion Kit Sigma). The same plasma volume equivalent (50 µL) was used for the depletion of all samples. Depleted fractions were aliquoted and stored at −80 °C. Protein concentration was determined after thawing using the Bradford assay.

The brain samples (n = 2) were obtained from The Netherlands Brain Bank, Netherlands Institute for Neuroscience, Amsterdam (open access: www.brainbank.nl). Donors provided a written informed consent for a brain autopsy and the use of the material. For one of the subjects, TDP-43 positive inclusions were reported. Brain tissues were homogenized in 8 M urea, 75 mM NaCl, 50 mM Tris, pH 8.2 with protease and phosphatase inhibitors (Roche).

### Digestion, TMT 10 labeling and clean-up

100 µg total proteins per sample (depleted plasma and brain lysates) were processed as previously described^17^. 10 µg of six depleted plasma were mixed with brain lysates (105 µg) to generate a TMT 10plex (or Set). In total, five plexes were prepared. Figure S3 illustrates the experimental study design.

### SCX fractionation

The five TMT 10plexes were separated into 10 fractions by strong cation exchange (SCX), (polySULFOETHYL-A column (PolyLC)) on HPLC system (Waters Alliance 2695).

### MS analysis and computational MS

#### PBMC discovery MS method

Samples (1 µg) were analysed by LC-MS/MS using the EASY-nLC 1000 system coupled to an Orbitrap Fusion™ Tribrid™ mass spectrometer (both Thermo Scientific). Dried samples were reconstituted in 2% ACN and 0.1% formic acid (FA) in water. Re-suspended peptides were loaded on a column packed with C18 reverse phase. 180 min gradient method ranging from 8% of ACN in 0.1% FA/water to 100% of ACN at a flow rate of 200 nL/min was used. MS spectra ranging from 400 to 1,400 m/z were acquired at resolution settings of 120,000 and 30,000 for full-scan MS and MS2, respectively.

#### Plasma and brain discovery MS method

Samples (4 µg) were analysed by LC-MS/MS/MS using the EASY-nLC 1000 system coupled to an Orbitrap Velos Pro Mass Spectrometer (both Thermo Scientific). Dried samples were reconstituted in 2% ACN and 0.1% FA in water. Re-suspended peptides were loaded onto a 2 cm long (OD 360 µm, ID 100 µm) capillary column filled with 5 µm ReproSil-Pur C18-AQ (Dr. Maisch GmbH) and resolved using an increasing gradient of ACN in 0.1% formic acid through a 15 cm long (OD 360 µm, ID 75 µm) capillary column filled with 3 µm ReproSil-Pur C18-AQ (Dr. Maisch GmbH) at a flow rate of 200 nL/min.

Peptide mass spectra were acquired throughout the entire chromatographic run (115 minutes) using TOP 10 collision induced dissociation (CID) method enabling higher collision induced dissociation (HCD) MS3 scans for accurate quantitation of TMT reporter ions. Due to the high complexity of proteins/peptides within the given matrix, MS3 method was chosen rather than the MS2 method. The isobaric quantitation methods are prone to interference of co-isolating peptides and MS3 eliminates ratio distortion in isobaric multiplexed quantitative proteomics^38^. The MS3 method omits this bias by allowing a higher accuracy of the observed quantitation ratios. Each sample was analysed by two LC-MS/MS analytical repeats.

### Computational MS

Raw MS data files were submitted to Proteome Discoverer (PD) v1.4 (Thermo Scientific). Each raw file was searched as individual file on Proteome Discoverer 1.4 using a human FASTA file data base (UniProtKB database; March 2015). Results from SEQUEST searches were exported with 1% FDR & ≥1 rank one peptides per protein settings.

### Bioinformatics

#### Data assembly and normalisation

PBMC discovery: Proteome Discoverer output files were filtered to include peptide spectral matches (PSMs) with signal in at least 1 out of 10 TMT reporter ion channels. TMT reporter ion intensities were normalised using sum-scaling. All reporter ion intensities were summed and the median value across all summed channels was calculated. A correction factor was obtained by dividing the individual summed reporter ion intensity for a channel by the median of all channels. Each PSM ion intensity was multiplied by the correction factor for the relevant TMT reporter channel. After sum scaling, log2 ratios of each PSM were calculated relative to the median of 10 channels. The PSM level ratios were then summarised to peptide level ratios as an average across the PSM level ratios specific to a peptide sequence. In the second stage of data processing, peptide features with more than ~66% missing quantitative values in clinical groups were removed from the data set. The remaining missing quantitative values were replaced by values imputed using the k-nearest neighbour imputation method which was applied to the samples from the same experimental group. Data were normalised across the TMT 10plex sets using quantile normalisation method. To reduce the influence of technical factors, a batch effect removal procedure was applied. For protein-level analysis, expression values were computed by averaging peptides unique to a gene identifier.

Plasma and brain discovery: For all PSM’s, the reporter intensities in all channels were background and isotopically corrected in order to remove cross-talking between channels. Then, reporter ion intensities of six plasma samples (126, 127 N, 127 C, 128 N, 128 C, 129 N) per TMT 10plex experiment were calibrated based on the reference intensity distribution extracted from four brain calibrant samples (129 C, 130 N, 130 C, 131). This was achieved by scaling of these four channel distributions to the median intensity of the lowest intensity channel (129 C), then scaling each calibrator channel to the same median intensity. The resulting median PSM intensity value was used for the calculation of the ratios of plasma sample. These calibrated intensities were log2-transformed. PSMs having no quantitative data were discarded. To generate a peptide matrix from all PSMs, the median of log2-ratios was used to calculate calibrated quantitative peptide data. Following this step, the data was normalised by the median normalisation method. Afterwards, peptide features with more than ~35% missing quantitative values in clinical groups were removed from the data sets. The remaining missing quantitative values were replaced by values imputed using the knn = 2 imputation method, applied to samples from the same treatment group. To reduce the influence of technical factors, a batch effect removal procedure was applied. For protein-level analysis, expression values were computed by averaging peptides that were unmodified and unique to the gene identifier.

### Principal component analysis

PCA scores and loading plots were generated, at peptide and protein levels, to study the variance structure of the data sets. PCA was performed after each step of data treatment (e.g. batch effect removal, normalisations) to identify technical and sample inherent factors influencing the data variance. The scores plots enabled visualisation of the experimental groups and an alternative means of assessing whether any samples could be considered as outliers, given their position with respect to other samples from the same class and the confidence interval ellipse displayed (80%). The loadings plots allowed assessment of the magnitude and correlation of effects.

### Feature selection

Linear modelling using the R package LIMMA was applied to perform a multivariate statistical analysis to identify regulated peptides and proteins. The experimental group was analysed using the following multivariate linear model: logRatio ~0 + Group. Log2fold change (log2FC) and p-values were calculated for all features (peptides and proteins). The significance criterion α was standardly set = 0.05 and a log2-fold change threshold = 1.3 was required to consider a feature as “regulated”. Multiple testing corrections were performed using the Benjamini-Hochberg procedure.

### Functional analysis

Functional analysis was performed by an in-house tool (Functional Analysis Tool; FAT) to explore the significance of regulation analysis, evaluated by the Mann-Whitney U test. Functional analysis was performed to indicate pathways affected when comparing B-ALS versus L-ALS. For the regulation analysis, a two-sided p-value was generated by the Mann Whitney U test and the Benjamini-Hochberg method was used for multiple test correction. In this procedure, expression values for all terms were compared to a background composed of 1000 randomly-sampled expression values that did not overlap with the term. A minimum of three matched identifiers (e.g. gene names) was required. Terms with an adjusted p-value < 0.05 were considered significant. All functional results were visualised in volcano plots (median fold change vs. adjusted p-value). Full results, with matched gene names, are provided in.csv format.

### Immonodetection by MSD and Simoa

The quantitative determination of NfL and NfH in human plasma was performed by Single Molecule Array (Simoa) technology using a digital immunoassay Simoa HD-1 Analyzer; (Quanterix, Lexington, MA).

Expression analysis of apolipoprotein E protein (APOE; R-PLEX Antibody Set F212I) was carried out by enzyme-linked immunosorbent assay (ELISA) using an electrochemiluminescence (ECL)-based Meso Scale Discovery (MSD) platform.

Standards, primary and secondary antibodies, detection range including lower and upper limits of detection were specified in the manufacturer’s conditions of the commercial assays.

Plasma samples from ALS patients and healthy patients were equally distributed on each plate and measured in duplicate.

### Statistical analysis Re-Test NfL, NfH and APOE

For the immunoassay data, statistical analysis was performed using GraphPad Prism 6. Continuous variables were analysed by one-way Anova (Kruskal-Wallis) nonparametric analysis for group comparisons (Dunn’s multiple comparisons test). Receiver operating characteristic curve analysis was used to assess assay sensitivity/specificity and diagnostic performance. A p-value < 0.05 was considered statistically significant.

## Supplementary information

Supplemental Data

Plasma_peptides

Plasma_proteins

PBMC_peptides

PBMC_proteins
