# Author Correction: Combined Tissue-Fluid Proteomics to Unravel Phenotypic Variability in Amyotrophic Lateral Sclerosis

**DOI:** 10.1038/s41598-020-74974-1

**Published:** 2020-10-23

**Authors:** Emanuela Leoni, Michael Bremang, Vikram Mitra, Irene Zubiri, Stephan Jung, Ching-Hua Lu, Rocco Adiutori, Vittoria Lombardi, Claire Russell, Sasa Koncarevic, Malcolm Ward, Ian Pike, Andrea Malaspina

**Affiliations:** 1 Proteome Sciences R&D GmbH & Co. KG, Altenhöferallee 3, 60438 Frankfurt am Main, Germany; 2 Proteome Sciences plc, Hamilton House, Mabledon Place, London, WC1H 9BB UK; 3 Centre for Neuroscience and Trauma, Blizard Institute, Queen Mary University of London, 4 Newark Street, London, E1 2AT UK

Correction to: *Scientific Reports* 10.1038/s41598-019-40632-4, published online 14 March 2019

The link to the original data is incomplete. The following Data Availability text should be included:

“The mass spectrometry proteomics data have been deposited to the ProteomeXchange Consortium via the PRIDE partner repository with the dataset identifier PXD020493.”

