# Supplementary material for: Combined Tissue-Fluid Proteomics to Unravel Phenotypic Variability in Amyotrophic Lateral Sclerosis
Source: Sci Rep. 2019 Mar 14;9:4478. doi: 10.1038/s41598-019-40632-4 (PMC6418138; doi:10.1038/s41598-019-40632-4)
Supplement: Supplementary file 1 — Supplemental Data [file 41598_2019_40632_MOESM1_ESM.doc]

**Combined tissue-fluid proteomics to unravel the phenotypic variability in amyotrophic lateral sclerosis**

Emanuela Leoni^a^, Michael Bremang^b^, Vikram Mitra^b^, Irene Zubiri^c^, Stephan Jung^a^, Ching-Hua Lu^c^, Rocco Adiutori^c^, Vittoria Lombardi^c^, Claire Russell^b^, Sasa Koncarevic^a^, Malcolm Ward^b^, Andrea Malaspina*^c^, Ian Pike*^b^

## Supplemental Data


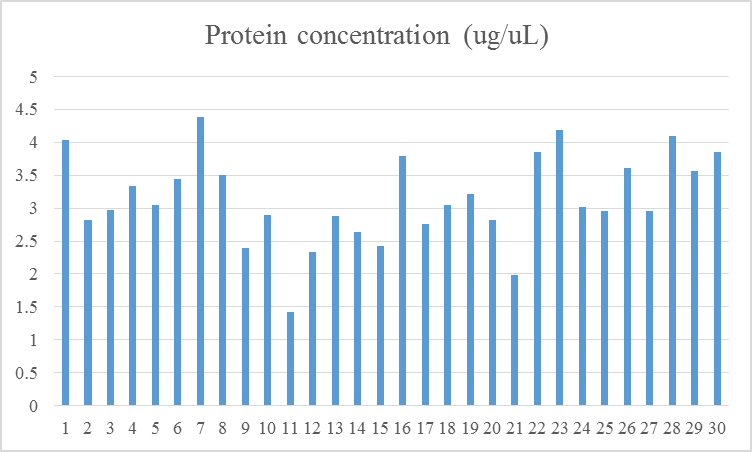
Figure S1. Plasma sample characteristics. Protein concentration in all 30 samples determined by the Bradford assay after albumin and IgG depletion. The average protein concentration after depletion was 3.14 µg/µL (mean value), with B-ALS having a higher total protein content compared to L-ALS (3.3 µg/µL versus 2.9 µg/µL).


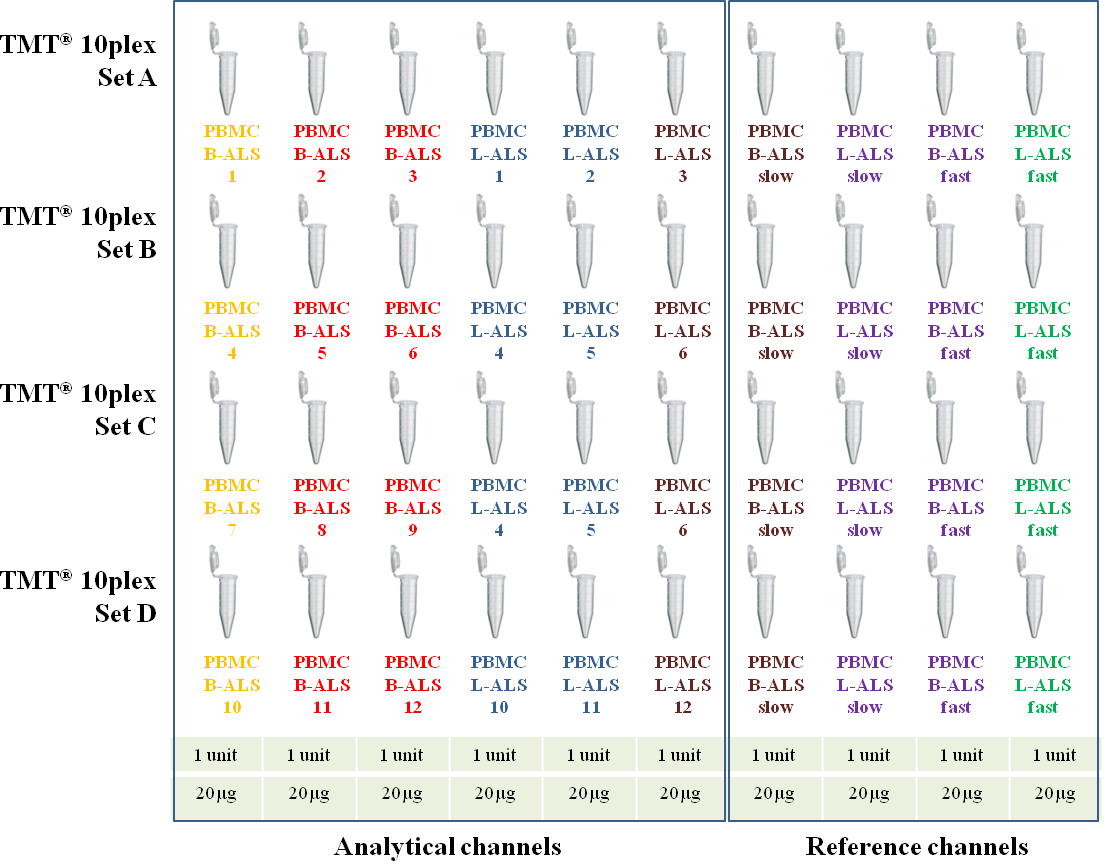


Figure S2. Study design of the PBMC proteomic study. In each TMT 10plex, six channels were allocated for analytical samples (PBMC lysates) and four channel for the reference samples (mixture of the different patients’ groups). B-ALS (n=12) and L-ALS samples (n=12) were analysed in four independent TMT 10plexes (Set A to D). The color code represents the TMT reagent used for corresponding sample type (i.e. yellow: 126, red: 127N and 127C, blue: 128N and 128C, brown: 129N and 129C, purple: 130N and 130C, green: 131).


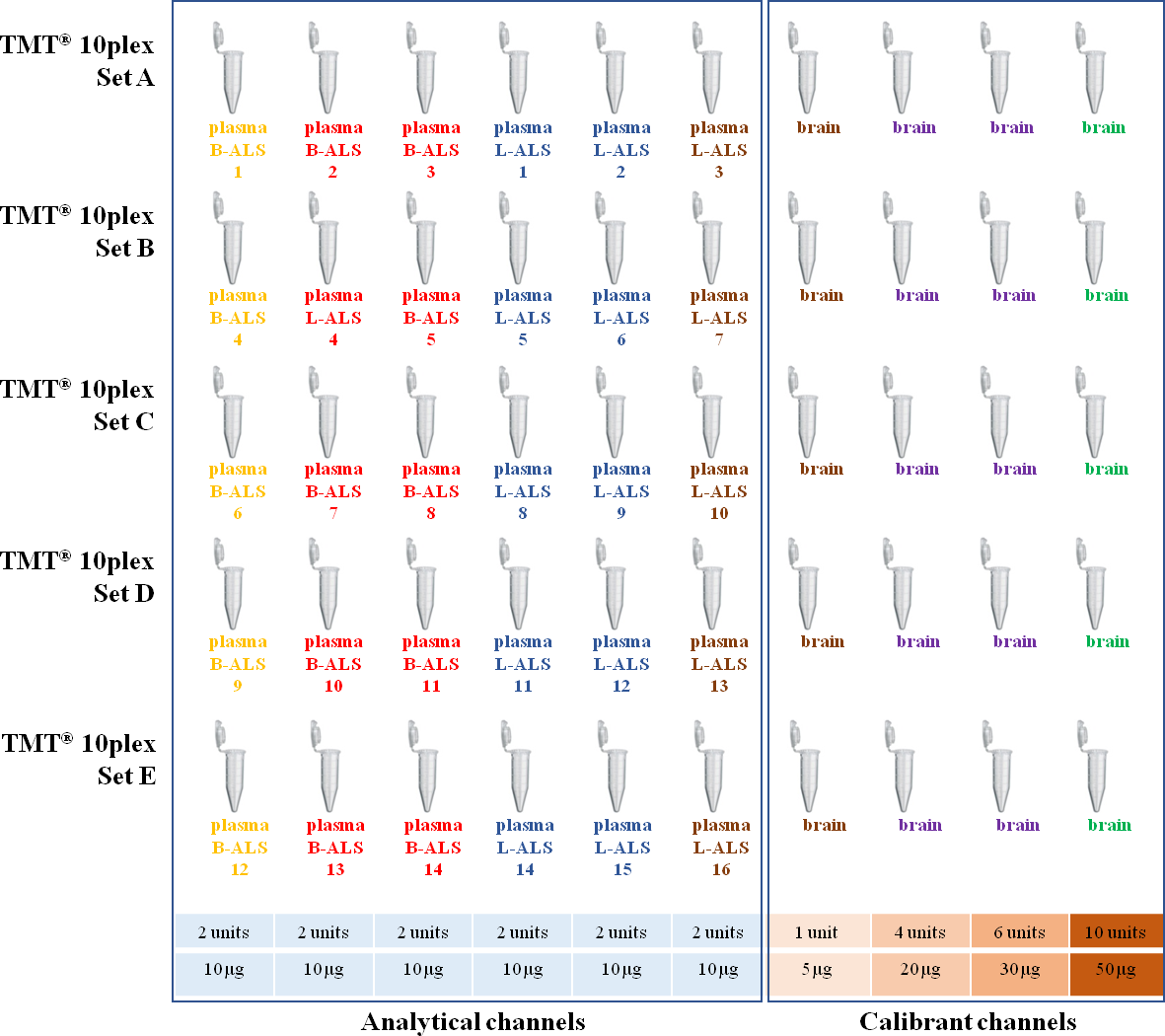
Figure S3. Study design of the TMTcalibrator plasma/brain study. In each TMT 10plex, six channels were allocated for analytical samples (albumin and IgG-depleted plasma samples) and four channels for the calibrant samples (brain lysates) in different amounts (1-4-6-10 units) to trigger the detection of low abundant brain-derived proteins in the plasma. B-ALS (n=14) and L-ALS samples (n=16) were analysed in five independent TMT 10plexes (Set A to E). Patient L-ALS 6 was discarded during the data processing and statistical analysis due to the lack of clinical information (diagnostic latency, spasticity scale, cognitive impairment, riluzole). The color code represents the TMT reagent used for corresponding sample type (i.e. yellow: 126, red: 127N and 127C, blue: 128N and 128C, brown: 129N and 129C, purple: 130N and 130C, green: 131).


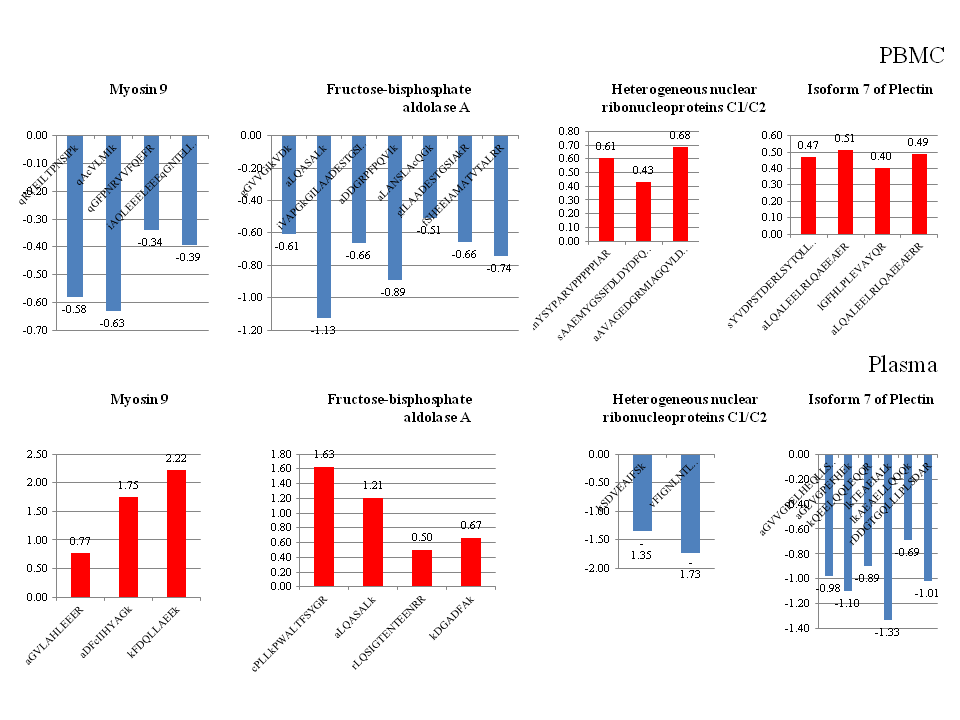
Figure S4. Protein of interest whose peptides were significantly regulated in both the two matrices.


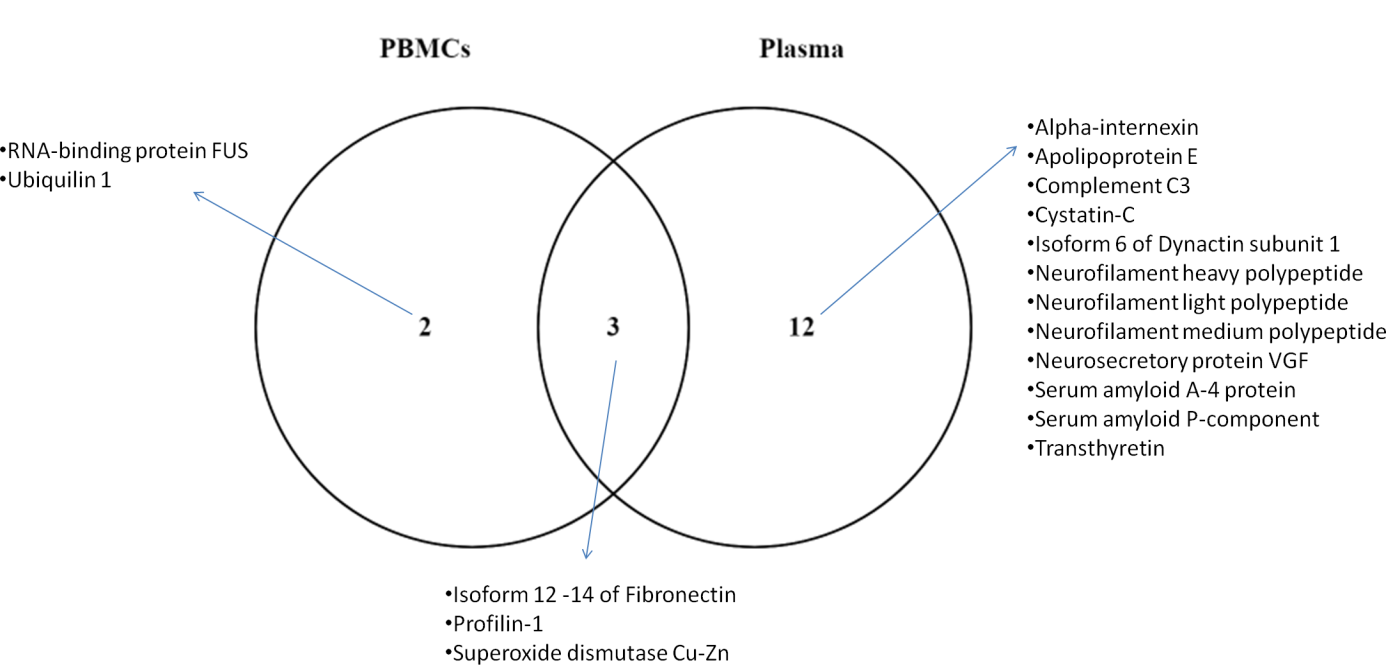


Figure S5. ALS proteins identified in the PBMC and plasma/brain proteomic studies when comparing B-ALS versus L-ALS. Proteins reported in PBMC and plasma only compartments of the Venn diagram are protein candidates already identified as linked to the pathogenesis of ALS. RNA-binding protein FUS and Superoxide dismutase Cu-Zn were found significantly regulated (p-value <0.01) in PBMC at protein level. Alpha-internexin, complement C3, neurofilament light polypeptide, neurofilament medium polypeptide, profilin-1, serum amyloid P-component and transthyretin were found significantly regulated (p-value <0.01) in depleted plasma at protein level.


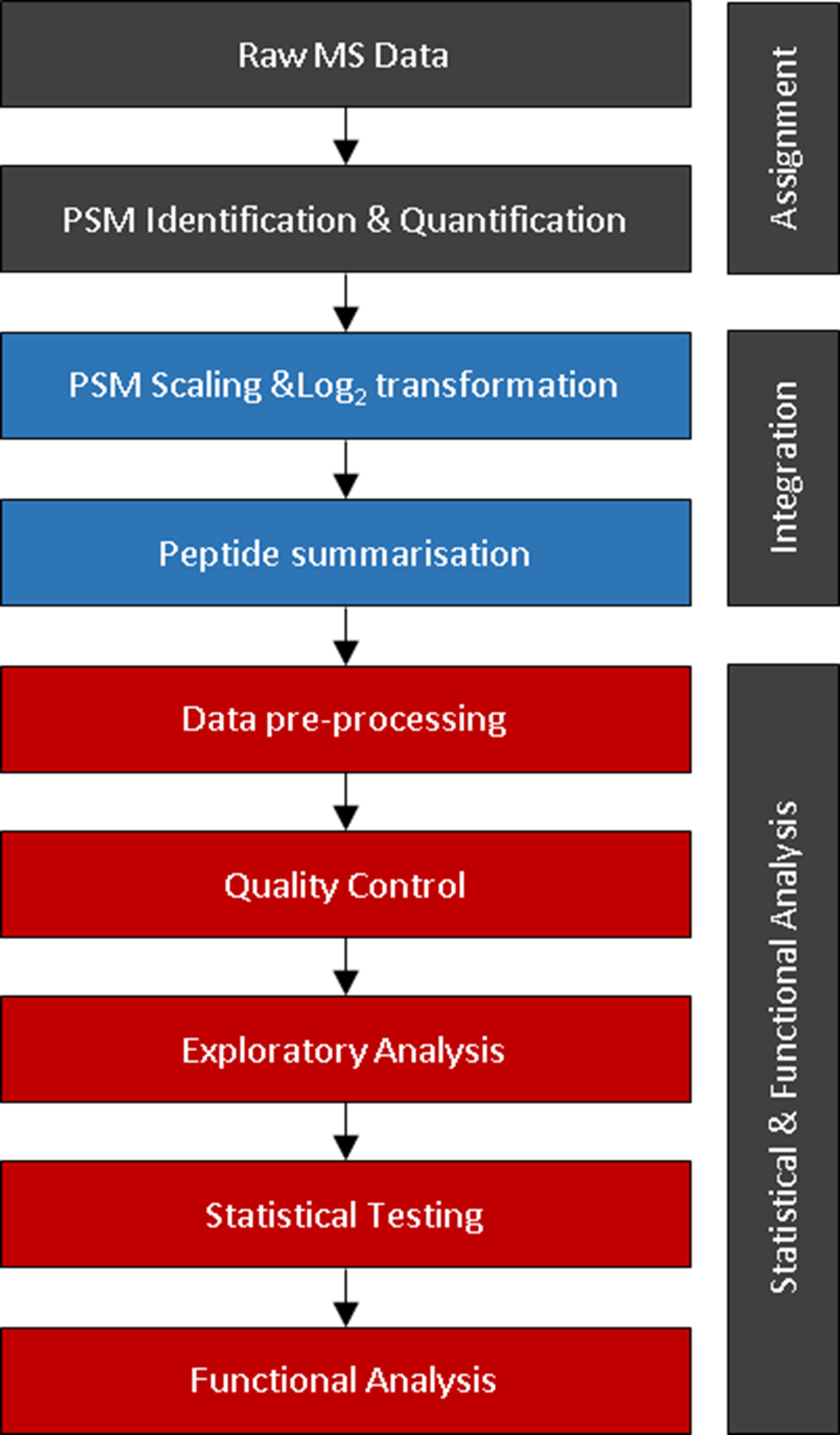


Figure S6. Flowchart describing the overall bioinformatic pipeline.

| **Sample ID** | **number cells/sample** | **volume of lysis buffer used (μL)** | **protein concentration (μg/μL)** |
| --- | --- | --- | --- |
| B-ALS slow |  |  |  |
| B-ALS 1 | 5x10^6 | 600 | 0.3 |
| B-ALS 2 | 0.9x10^6 | 300 | 0.7 |
| B-ALS 3 | 4.6x10^6 | 600 | 0.5 |
| B-ALS 4 | 3.3x10^6 | 500 | 0.6 |
| B-ALS 5 | 1.3x10^6 | 400 | 0.1 |
| B-ALS 6 | 0.9x10^6 | 300 | 0.6 |
| L-ALS slow |  |  |  |
| L-ALS 1 | 2.5x10^6 | 500 | 0.6 |
| L-ALS 2 | 4.6x10^6 | 600 | 0.3 |
| L-ALS 3 | 2.2x10^6 | 500 | 0.4 |
| L-ALS 4 | 1.6x10^6 | 400 | 0.3 |
| L-ALS 5 | 1.25x10^6 | 400 | 0.2 |
| L-ALS 6 | 4.3x10^6 | 600 | 0.4 |
| B-ALS fast |  |  |  |
| B-ALS 7 | 2.9x10^6 | 500 | 0.7 |
| B-ALS 8 | 3.4x10^6 | 500 | 0.5 |
| B-ALS 9 | 2.4x10^6 | 500 | 0.4 |
| B-ALS 10 | 2.2x10^6 | 500 | 0.5 |
| B-ALS 11 | 5.1x10^6 | 600 | 0.3 |
| B-ALS 12 | 4.6x10^6 | 600 | 0.4 |
| L-ALS fast |  |  |  |
| L-ALS 7 | 4.5x10^6 | 600 | 0.6 |
| L-ALS 8 | 0.4x10^6 | 300 | 0.2 |
| L-ALS 9 | 9.68x10^6 | 1000 | 0.5 |
| L-ALS 10 | 3.5x10^6 | 500 | 0.9 |
| L-ALS 11 | 2.1x10^6 | 500 | 0.1 |
| L-ALS 12 | 5.4x10^6 | 600 | 0.8 |

Table S1. PBMC sample characteristics (number of cells/samples, volume of urea buffer and total protein concentration). The average protein concentration in PBMC lysates was 0.44 µg/µL with B-ALS having slightly higher concentration compared to L-ALS (0.46 µg/µL *versus* 0.4 µg/µL). Samples from fast progressing patients had higher concentration compared to slow progressing (0.5 µg/µL *versus* 0.4 µg/µL). Total mass of proteins (µg) was highly correlated with the initial number of cells.
